# Leadership and management curriculum planning for Iranian general practitioners

**Published:** 2015-10

**Authors:** SHAHLA KHOSRAVAN, HOSSEIN KARIMI MOONAGHI, SHAHRAM YAZDANI, SOLEIMAN AHMADI, MOHAMMAD REZA MANSOORIAN

**Affiliations:** 1 Department of Community & Mental Health Nursing, Social Determinants of Health Research Center, Gonabad University of Medical Sciences, Gonabad, Iran;; 2 Department of Medical-Surgical Nursing, Nuking & Midwifery School, Mashhad University of Medical Sciences, Mashhad, Iran;; 3 Department of Medical Education, School of Medical Education, Shahid Beheshti University of Medical Sciences, Tehran, Iran;; 4 Faculty of Medicine, Tehran University of Medical Sciences, Tehran, Iran

**Keywords:** Curriculum, Management, Medical education, General practitioner

## Abstract

**Introduction:**

Leadership and management are two expected features and competencies for general practitioners (GPs). The purpose of this study was leadership and management curriculum planning for GPs which was performed based on Kern’s curriculum planning cycle.

**Methods:**

This study was conducted in 2011- 2012 in Iran using an explanatory mixed-methods approach. It was conducted through an initial qualitative phase using two focus group discussions and 28 semi-structured interviews with key informants to capture their experiences and viewpoints about the necessity of management courses for undergraduate medical students, goals, objectives, and educational strategies according to Kern’s curriculum planning cycle. The data was used to develop a questionnaire to be used in a quantitative written survey. Results of these two phases and that of the review of medical curriculum in other countries and management curriculum of other medical disciplines in Iran were used in management and leadership curriculum planning. In the qualitative phase, purposeful sampling and content analysis with constant comparison based on Strauss and Corbin’s method were used; descriptive and analytic tests were used for quantitative data by SPSS version 14.

**Results:**

In the qualitatively stage of  this research, 6 main categories including the necessity of management course, features and objectives of management curriculum, proper educational setting, educational methods and strategies, evolutionary method and feedback result were determined. In the quantitatively stage of the research, from the viewpoints of 51.6% of 126 units of research who filled out the questionnaire, ranked high necessary of management courses. The coordination of care and clinical leadership was determined as the most important role for GPs with a mean of 6.2 from sample viewpoint. Also, team working and group dynamics had the first priority related to the principles and basics of management with a mean of 3.59. Other results were shown in the paper.

**Conclusion:**

Results of this study indicated the need to provide educational programs for GPs; it led to a systematic curriculum theory and clinical management using Kern cycle for general practitioner's discipline. Implementation and evaluation of this program is recommended.

## Introduction

Objectives and functions of healthcare system and clients’ needs and demands are variable and influence one another over the time. These two factors have direct impacts on duties and roles of GPs (1). Development and complexity of healthcare system has created more opportunities for leadership and management (2).

Physicians having the power of management are important elements in the success of healthcare system and as managers and leaders create a dynamic balance between authority, accountability and control of input, throughput, and output in team working. Physicians are often asked to undertake managerial roles in health organizations or educational groups; thus, in medical education training program basic concepts such as the structure of team working and the meaning of leadership must be included. They have to possess knowledge and necessary skills in group dynamics and leadership to enable them to manage complicated systems (3). To respond to real needs of modern societies, training effective human resources is the duty of educational systems and universities (4).

Complex challenges and medical features necessitate attention to leadership and management skills whereas little attention has been paid to the traditional medical education and teaching formal experience in the world and in Iranian medical colleges (5).

The necessity of a reform in medical education by scholars and medical education planners has been posed in the world (6). The medical society of America has considered leadership development as one of the main educational missions (7). Educational content of general practitioner curriculum must have a dynamic approach (5). So that these graduates could do their duties well in future (8). This involves a reform in medical education curriculum to fulfill those needs (9).

Although there have been some limited surveys in other countries, the modality of implementing this course and educational needs of students in general practitioner’s field has been assessed. However, a comprehensive curriculum plan for management and leadership in GPs’ discipline in Iran and some other countries has not been taken into account (8-16). This study was performed based on the first fourth phases of  Kern’s cycle curriculum planning model (problem identification and general needs assessment, needs assessment of target  learners, setting goals, and educational strategies) to develop leadership and management course in Iran’s general practitioner training.

## Methods

This study used an explorative mixed method based on needs assessment which was performed in 2011-2012 in Shahid Behshti University of Medical Sciences in Iran. In this study, data collection was carried out in two qualitative and quantitative stages.

According Kern’s curriculum planning cycle, the experts’ views  and experiences about the necessity of management courses for undergraduate medical students, goals, objectives, and education strategies were required; therefore, by purposeful sampling in the first stage, deep and comprehensive data were gathered through two focus group discussions and 28 semi-structured interviews with key informants in healthcare management and medical education, physician directors and organizational colleagues such as nurses, administrative and insurance experts. Semi-structure interviews and focus group discussion were conducted with participants who were experts in management and medical education. The data of this phase was analyzed with constant comparison based on Strauss and Corbin’s method. Trustworthiness of the data analysis was established by quality method such as checking inter-rater reliability (at least 80%) and member checking (11). Therefore, the qualitative data and also data captured from review of medical fields in Iran and management and other leadership curricula of general practitioner and medical specialties in other countries were used to provide a questionnaire. The survey questionnaire had two sections. The first section was used to generate demographic information. The second section was a 5-point Likert-type scale, which was used to assess the subjects’ viewpoints about the necessity of management courses, issues, content and objectives, educational methods and strategies, implementation and educational settings, evaluation methods and feedbacks. The participants were asked to select one of the five possible choices from high necessary (4) to unnecessary (0).

The validity of the above scale was determined through content validity (approval of experts’ view) and reliability by Cronbach’s Alpha coefficient (0.73). 

In the second stage, in a quantitative survey, this questionnaire was filled out by 126 samples based on target and stratified random sampling method. The subjects of this study consisted of management science experts, medical education faculty members, physician managers and their colleagues. The study setting consisted of virtual domain, manuals, Iran’s medical universities, and Iran’s Ministry of Health, workplace of GPs, medical council, and insurance companies. Descriptive statistics were used to summarize the quantitative data from the survey instrument. To do so, SPSS software 14 was used. The level of significance was set at below 0.05.

This study was approved in educational development center of Shahid Beheshti University of Medical Sciences. The ethical considerations in this study were data collection through informed consent of the subjects and the possibility of exclusion from this plan. Additionally, giving importance to all their viewpoints and trustworthiness has been observed and the results of this survey were scientifically published for stakeholders. 

## Results

The qualitative data were gathered from two focus group discussions with 14 participants and 28 semi-structured interviews with 24 participants (totally 38 participants). The necessity of management courses for general practitioners along with the knowledge and skills required, features and objectives of management curriculum, proper educational setting, educational methods and strategies, evolutionary method and feedback result was determined from the first qualitative step of research (11).

In the second stage of the research, on the whole, 126 completed questionnaires were received. (38.1% female, 61.9% male). 7.1% had bachelor degree, 15.9% had master's degree, 37.3% were general practitioners, 20.6% were specialists and 19.0% had Ph.D. Their fields of study were management (5.6%), general practitioners (36.5%), medical education (13.5%), nursing (9.5%), midwifery (2.3%), social physician (15.1%), internal medical specialist (5.6%), surgical specialist (9.5%) and other relevant medical fields of study (1.6%). 39.7% of the samples besides their initial fields of study had MS degree in medical education. 

85.7% had some experience in organizational management, and 98.4% had directly worked with physicians training management for GPs. 

51.6% of the samples ranked the management courses as the high necessary, 32.5% necessary, 13.5% rather high, and only 2.4% low necessary.

Delegating the authority of management of organizations to GPs from the viewpoints of samples was as follows: 81% of managers of health organizations, 84.9% of treatment organizations, 56.3% of educational and cultural organizations, and 11.9% of managers of financial administrations.

Ranking of GPs’ role in management is shown in Table 1. As shown, the most important role of general practitioners is coordination of care and clinical leadership (Table 1).

**Table 1:** Ranking of GPs’ role in management

**Rank**	Role	Mean score(0-4)
**1**	Coordinator of care and clinical leadership	6.27
**2**	Community leader for control determent of health	4.07
**3**	Health risk management	3.96
**4**	Administrator role in health system management	3.84
**5**	Health policy analysis	3.47
**6**	Disease management in target population	3.16
**7**	Fund holding	3.03

The prioritization of ranking of issues related to the principles and basics of management is shown in Table 2. According to this table, team working and group dynamics had the first priority.

**Table 2:** The prioritization of ranking of issues related to the principles and basics of management

**Rank**	Issus	Score (0-4)
**1**	Team working & group dynamics	3.56
**2**	Risk management & problem solving	3.48
**3**	Successful time management	3.45
**4**	Management & leadership	3.43
**5**	Assessment & evaluation	3.39
**6**	Organizational behavior & stress management	3.34
**7**	Planning	3.32
**8**	Organizing	3.30
**9**	Decision making & ranking goals	3.29
**10**	Quality management	3.28
**11**	Supervision & control	3.27
**12**	Motivation	3.26
**13**	Guidance and counseling	3.23
**14**	Management process	3.22
**15**	Co-ordination	3.21
**16**	Communication and meeting management	3.17
**17**	Authorities and responsibility	3.13
**18**	Effectiveness & efficiency	3.12
**19**	Reform & change management	3.10
**20**	Budgeting	2.86
**21**	Management theory & systematic thinking	2.77

The ranking of healthcare and health system management issues in management course are shown in Table 3. As shown, health promotion is of prominent importance among the proposed issues for the course of management health system.

**Table 3:** The ranking of healthcare and health system management issues in management courses

**Rank**	Health care management issues	Mean score (0-4)
**1**	Health promotion	3.60
**2**	Delivering services (ambulatory, inputting, public health, health promotion)	3.59
**3**	Hospital organization	3.41
**4**	Responsiveness in healthcare system	3.40
**5**	Health care system in Islamic Republic of Iran	3.39
**6**	Organization of health centers	3.38
**7**	Organization of health network	3.36
**8**	Health system functions	3.33
**9**	Organization ministry of health & Medical education	3.28
**10**	Medical university & health service duties	3.23
**11**	Health house function	3.22
**12**	Goals achievement	3.21
**13**	Stewardship (policy making, intra-spectral governance, intra-spectral leadership)	3.16
**14**	Creating resources (input management, human recourse, pharmaceuticals technology, consumables capital)	3.15
**15**	Fair financial contributions	3.13
**16**	Insurances	3.12
**17**	Private office & hospital clinic	3.11
**18**	Medical council	3.10
**19**	Financing (revenue collection, pooling, allocate purchasing)	3.03

Issue of health promotion with a mean score of 3.6 from 4 achieved the highest priority.

The characteristics of management course in curriculum planning for GPs are shown in Table 4. According this Table, 70% of the samples believed that each theory course of principles and basics of management and health system management should change from 17 to 34 hours, but management training in field should change from 70 to 140 hours. Other relevant data are displayed in Table 4. Results from the qualitative phase were reported in first publication of this study (11).

**Table 4:** Characteristics of the management course in GPs curriculum plan

**Instructor specialty**	**Student assessment** **method**	**Teaching method**	**Educational strategy**	**Course objectives**	**Place of presentation of course**	**Presentation** **time**	**Number of units**	**Course** **type**	**Course title**	**Sequence of presentation**
**Management and medical education**	Proper written tests, physical presence evaluation, punctuality and discipline, active participation scientifically and practically, doing the duties assigned	Lecture, heuristic methods, problem solving methods, active learning	Use of SPICES strategies	In accordance with curriculum	Class or conference hall	Fourth term or semester basic sciences	1 or 2 (preferably 2)	Theory	Principles and basics of management	**First**
**Healthcare management, medical education**	Proper written tests, physical presence evaluation, punctuality and discipline, active participation scientifically and practically, doing the duties	Lecture, heuristic methods, problem solving methods, active learning	Use of SPICES strategies	In accordance with curriculum	Class or conference hall	Physiopathology	1 or 2 (preferably 2)	Theory	Health system management	**Second**
**Healthcare management, social physician,** **medical education**	Interview, observation, log book, portfolio, check list	Clinical education, heuristic methods, problem solving methods and learning based on job tasks	Using SPICES strategies and learning based on lead up	According to curriculum and in accordance with tasks	Real setting for providing healthcare	Apprenticeship	1 or 2 (preferably 2)	Apprenticeship	Management apprenticeship	**Third**
**Healthcare management, social** **physician, medical education**	Interview, observation, log book, portfolio, check list, system assessment report	Education in field, apprenticeship & internship through, learning based on tasks and role playing	Using SPICES strategies and learning based on lead up	According to curriculum and in accordance with job tasks	Real setting for providing health services	Internship	1 or 2 (preferably 2)	Internship	Management internship	**Fourth**

## Discussion

This study was performed based on Kern’s curriculum planning cycle with the aim of leadership and management curriculum planning for Iranian student general practitioners.

As viewed by most of the samples, training courses on management are necessary in Iran. Other reserchers have referred to general practitioner’s management roles originating from reform in health systems in other countries (14, 15) and have emphasized the importance and need for training courses in management in general practitioner’s curriculum (10, 11, 16).

From among these 7 leadership roles for general practitioners, the role of coordinator of care and clinical leadership is of vital importance which relates to the primary purpose of medical education, i.e. diagnosis and treatment of diseases (10). From among 21 presented issues in principles and basics of management, team working and group dynamics had the first priority. With respect to the effects of team working on clients, this issue has been considered suitable (17-19). To promote the professional behavior of physicians, creating group dynamics is important (10, 20).

In other studies, this issue has been shown to have a special importance (10, 21, 22).  From among 19 necessary issues in management of health system courses, 8 were of prime importance with an average of 3.6 is. Giving priority to this issue is beficial because paying attention to healthcare plans besides decreasing the disease is cost-effective (23, 24).

Suggested issues in these two theory course domains, “principles of management” and “management of health system research”, and its skills  do not completely conform with the performed needs assessment and residents’ training management (22), but our issues have a more coverage that may consequently be the reason for multi-source perspectives which most of them had passed; and also by working experience with physicians, they had conceived weaknesses in manager physicians who had not passed management course.

Taking into account management courses independently or integrated in GPs' curriculum in this and other studies has been emphasized (10, 1, 21, 25).

Based on the performed surveys by authors, often managerial issues for residents in the form of short-term courses or educational workshops are held (26, 27).

According to our research, few studies have assessed the quality and quantity of management courses in the form of theory and practice (12), similar to what was performed in this study. Suggested plans for this study are similar to management courses of other medical disciplines in Iran, such as nursing. Student-centered learning strategies recommended in this study are in the same line with other studies (10, 21, 28). But, teacher- centered learning in some of theory issues was proposed (11).

## Conclusion

Result of this study provide a framework for the development of four management curriculum courses for general practitioners including principles and basics of management, health system management, apprenticeship and internship management. These curricula were written on the basis of documents of expected competencies from alumni general practitioners of Islamic Republic of Iran for scientific management.

Although this curriculum was developed using a mixed method, limited sources of data were considered.  Implementation and evaluation of this curriculum and the impact of such a curriculum on leadership outcomes in the future are recommended. 
